# Simultaneous assay for protease activities of hepatitis C virus and human immunodeficiency virus based on fluorescence detection

**DOI:** 10.1038/s41598-019-45711-0

**Published:** 2019-06-24

**Authors:** Tsutomu Kabashima, Keiko Tonooka, Makoto Takada, Masaaki Kai, Takayuki Shibata

**Affiliations:** 1 Faculty of Pharmaceutical Sciences, Nagasaki International University, 2825-7 Huis Ten Bosch machi, Sasebo, Nagasaki 859-3298 Japan; 2 Department of Pathophysiology, Yokohama University of Pharmacy, 601 Matano-cho, Totsuka-ku, Yokohama-shi, Kanagawa 245-0066 Japan; 3 Faculty of Pharmaceutical Sciences, Graduate School of Biomedical Sciences, Nagasaki University, 1-14 Bunkyo-machi, Nagasaki, 852-8521 Japan; 4 Department of Laboratory Sciences, Gunma University Graduate School of Health Sciences, 3-39-22 Showa-machi, Maebashi-shi, Gunma 371-8514 Japan

**Keywords:** Biochemical assays, Proteases

## Abstract

Hepatitis C virus protease (HCV-PR) and human immunodeficiency virus protease (HIV-PR) are important for virus maturation, and thus can be used as potential target molecules for the development of antiviral drugs for the treatment of viral infections. In this study, a novel assay was developed to determine HCV-PR activity. This assay is based on a fluorogenic reaction, in which peptide fragments generated from an acetyl peptide substrate by HCV-PR can be selectively converted into a fluorescent derivative, and quantified by high-performance liquid chromatography (HPLC) with fluorescent detection. Herein, several acetyl-peptides can be used as substrates for HPLC. The application of this assay was further validated by simultaneous detection of HCV-PR and HIV-PR in a reaction mixture. The proposed method can differentiate the enzyme activities of HCV-PR and HIV-PR in a sample using their corresponding substrates. The results suggest that this assay can detect various proteases by employing set of substrate peptides under the same reaction conditions.

## Introduction

Since the identification of the hepatitis C virus (HCV) as the major pathogen responsible for parenterally transmitted non-A, non-B hepatitis^1^, chronic HCV infection has emerged as a global public health issue affecting approximately 170 million people worldwide. Patients with HCV infection often develop chronic hepatitis, which may lead to cirrhosis and hepatocellular carcinoma^2^, and are often co-infected with the human immunodeficiency virus (HIV). These HCV/HIV co-infected patients have graver liver-disease than patients infected with HCV alone^3^. Therefore, the HCV epidemic represents a huge unmet medical requirement for research efforts toward the development of better and convenient diagnostic tools.

HCV and HIV are single-stranded RNA viruses with a genome encoding a single polyprotein precursor. Maturation of the HCV polyprotein occurs through a series of proteolytic processes catalyzed by host cell proteases and the virally encoded protease^4^. The HCV NS3/4A protease (HCV-PR) as well as the HIV protease (HIV-PR) are thus important for virus replication, and molecules inhibiting them have been developed as anti-HCV and HIV drugs, respectively. Therefore, a selective and sensitive assay that can detect HCV-PR quantitatively or co-detect HCV-PR and HIV-PR is warranted for the screening of medicinal inhibitors.

High-performance liquid chromatography (HPLC)-based assays for protease activiy have been widely described^5^, in which various indicator moieties on synthetic peptide substrates have been developed. Fluorescence energy transfer (FRET)-based assays have also been intensively used to determine the activity of various proteases^6^. These assays employ a peptide substrate bearing a fluorescent moiety and a selected quenching moiety to allow continuous monitoring of protease activity. Enzyme-linked immunosorbent assays (ELISAs), in which the protease activity is determined by labeling amino moieties of the product with digoxigenin followed by an immunological reaction have also been reported^7^.

In this study, a simultaneous assay method was developed to determine the multi-enzymatic activities of HCV-PR and HIV-PR on the basis of a specific fluorescence (FL) reaction for a peptide that was previously reported^8^. This reaction would convert the C-terminal free peptide, catalyzed as a product by HCV-PR or HIV-PR, into fluorescent derivatives in the presence of catechol, borate, and sodium periodate, while the uncleaved acetyl substrate or its N-terminal acetyl fragment was not converted into fluorescent derivatives. The enzymatic activities of the proteases were detected by HPLC with FL detection. This assay was observed to be capable of sensitive quantification of the activities of HCV-PR and HIV-PR, and slso indicated that the lower detection limit of the enzymatic product was approximately 1.0 pmol/injection.

## Results

### Experimental design and optimization of FL reaction

**Figure 1:**
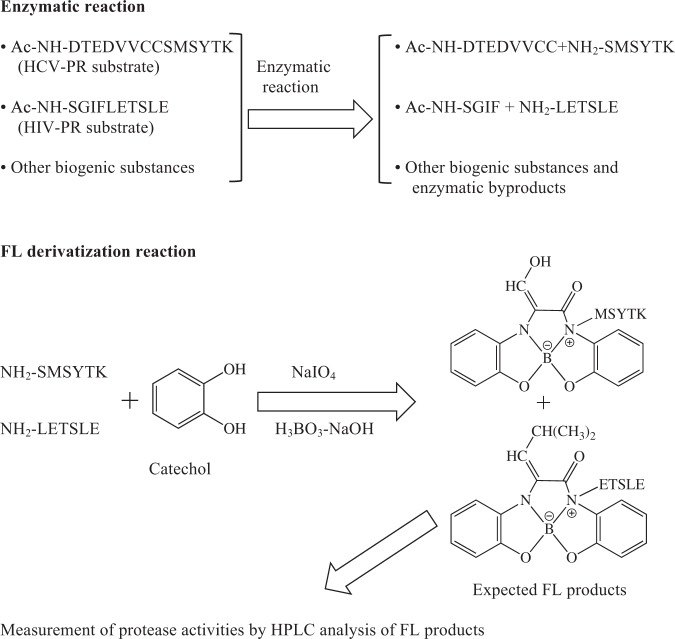
Schematic protocol for FL assay of HCV-PR and HIV-PR activities. The N-terminal acetyl peptides were used as substrates and specifically hydrolyzed by each protease. The generated peptides were selectively converted to the FL compounds by a fluorogenic reaction with catechol, sodium periodate, and borate. FL products were analyzed by HPLC with fluorescence detection.

FL-based techniques are widely applicable in protease activity determinations. We previously develop an assay for the quantification of HIV-PR activity using an FL reaction for a peptide^9^. In the present study, an FL reaction was used to assay HCV-PR activity. In the FL reaction, a peptide was converted into a fluorescent derivative in the presence of catechol, borate, and sodium periodate, but peptides with N-terminal acetyl groups did not produce this fluorescent derivative. On the basis of this result, an N-terminal acetyl peptide, Ac-DTEDVVCCSMSYTK was synthesized. This acetyl peptide contains a cleavage site of HCV-PR in the HCV polyprotein^6^, and was employed as a substrate. After enzymatic hydrolysis, the non-acetyl product, SMSYTK was selectively converted into a fluorescent compound, and monitored by HPLC with fluorescence detection, providing accurate quantitative information regarding HCV-PR activity (Fig. 1).

First, the FL reaction conditions were optimized using the synthetic peptide SMSYTK, which corresponds to the expected C-terminal cleavage product. The maximum yield of the FL product was obtained by heating the SMSYTK with catechol, sodium periodate, and sodium borate for 5 min at 100 °C in an aqueous solution (pH 7.0) as previously reported^9^. The FL products were found to be chemically stable at 4 °C for at least 48 h. To properly calculate the amount of the product generated from the substrates by enzymatic hydrolysis, a standard calibration curve was generated for the synthetic peptide SMSYTK in the concentration range of 0–20 μM. A linear correlation was obtained between the concentration of peptide and its fluorescent peak height on the HPLC chromatogram (Suppl. Fig. 1).

### Cleavage of the substrate peptide by HCV-PR

**Figure 2:**
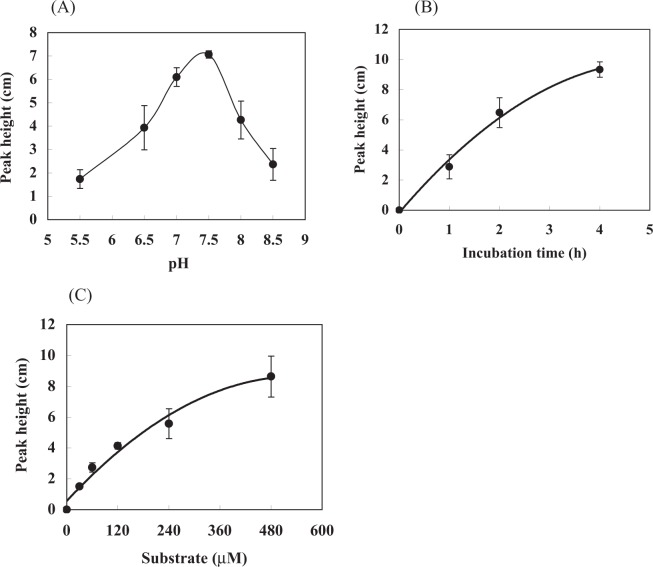
Effects of (**A**) buffer pH, (**B**) incubation time, and (**C**) substrate concentration on HCV-PR activity.

The influence of buffer pH and incubation time on the enzymatic reaction was examined. The results showed that HCV-PR cleaved the substrate most efficiently at pH 7.5 (Fig. 2A), and product yield increased with increasing incubation time up to 4 h (Fig. 2B). A pH 7.5 and an incubation time of 1 h were thus selected as the optimal enzymatic conditions for subsequent assays. Thereafter, the dependence of cleavage rate on substrate concentration was analyzed. The enzymatic reaction with HCV-PR was performed in a final volume of 20 μl of the reaction mixture. The HCV-PR (220 nM) was incubated with 0–480 μM substrate for 1 h, after which each fluorescent peak height at its corresponding substrate concentration was measured. As shown in Fig. 2C, the fluorescent peak height was approximately linear with substrate concentration up to 240 μM. On the basis of the above result, 240 μM substrate, within the linear range, in a 20 μL reaction mixture was used in all further experiments.

### Calibration curve for HCV-PR

**Figure 3:**
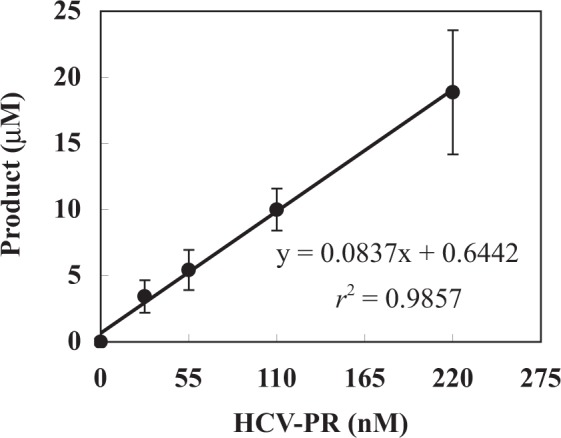
Calibration curve for HCV-PR.

A calibration curve for HCV-PR was investigated by the proposed method in the range of 0–220 nM in the enzymatic reaction mixture. As shown in Fig. 3, a linear relationship was observed between the HCV-PR concentration and the product concentration calculated from the fluorescent peak height (y = 0.0837x + 0.6442, *r*^2^ = 0.9857; x and y indicate the concentrations of HCV-PR and the enzymatic product, respectively). Therefore, the linear correlation between the HCV-PR concentration and the fluorescent peak height of the enzymatic product allows the measurement of HCV-PR activity.

A commercial kit, the SensoLyte 490 HCV Protease Assay Kit (AnaSpec, USA), was used to evaluate the reliability of the proposed method. The detection system of this kit was based on FRET using an FL-labeled substrate. The same amount of HCV-PR was used, and compared the peak height obtained from the proposed method with FL intensity from the kit. A good correlation (*r*^2^ = 0.98) of each activity of HCV-PR was obtained by both methods (Suppl. Fig. 2).

### Simultaneous assay of HCV-PR and HIV-PR activities

**Figure 4:**
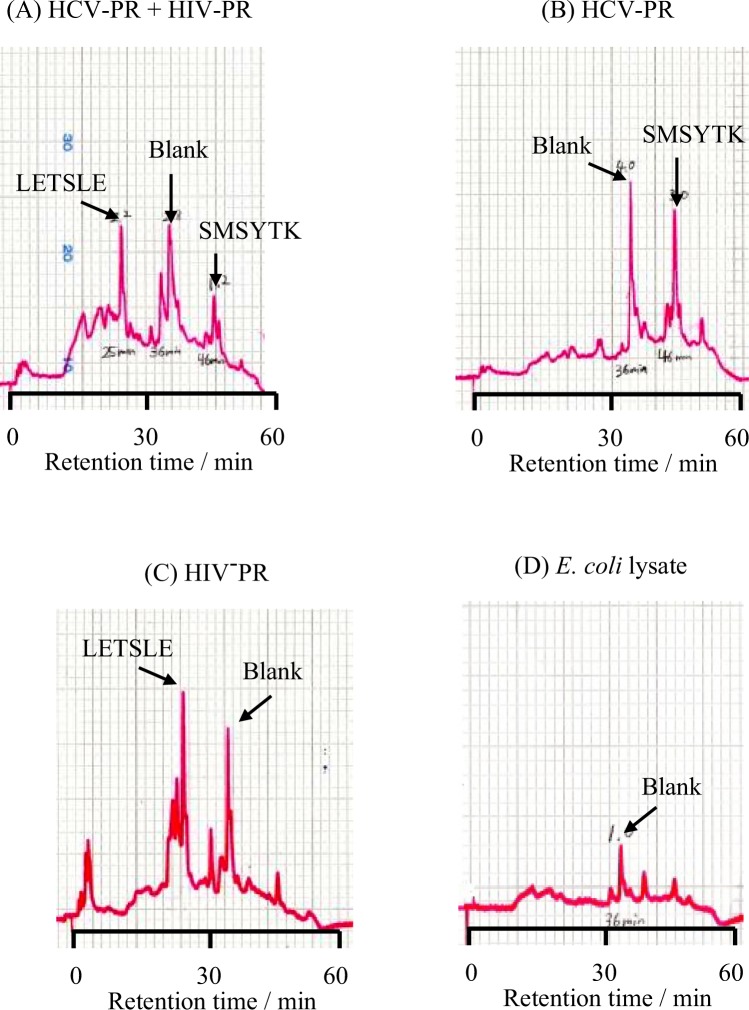
Simultaneous detection of HCV-PR and HIV-PR activities. A substrate mixture was incubated with (**A**) both HCV-PR and HIV-PR, (**B**) HCV-PR, (**C**) HIV-PR or (**D**) *E. coli* lysate without virus proteases. The enzymatic products of SMSYTK and LETSLE were converted to FL derivatives.

Co-infection with HCV is common among certain HIV-infected persons. Therefore, a selective and sensitive assay that can analyze the different virus proteases is desirable for therapeutic treatment and diagnosis. We previously developed the discrimination method of drug-resistant HIV mutants using several substrates^10^. Based on the report, additional acetylated peptide, Ac-SGIFLETSLE, was used as a substrate for HIV-PR. First, the separation of two synthetic peptides (LETSLE and SMSYTK) was examined. These corresponded to the products by enzymatic digestion (Fig. 1). These peptides could be separated and detected by HPLC, respectively (Suppl. Fig. 3). Discrimination of HCV-PR from HIV-PR was performed by incubating the two proteases in an enzyme-reaction mixture that contains the substrates for HCV-PR and HIV-PR, respectively. The substrate mixture was incubated with HCV-PR alone or with HIV-PR alone and these served as the control. As shown in Fig. 4A, C-terminal cleavage products (SMSYTK and LETSLE) enzymatically derived from the substrates could be derivatized and fluorometrically detected, when both the substrates in a mixture were incubated with HCV-PR and HIV-PR. When the substrate mixture was incubated with either HCV-PR or HIV-PR, each product was observed (Fig. 4B,C). While *Escherichia coli* lysate without HIV-PR was used as a sample, a significant peak could not be observed (Fig. 4D). These results indicated that each substrate was specifically digested by the corresponding viral protease, and the enzymatic products with N-terminal free amino-group were converted into FL derivatives. In Fig. 4, a blank peak was derived from reagents.

## Discussion

In this study, a facile and selective assay for the simultaneous detection of the enzymatic activities of HCV-PR and HIV-PR has been described. This assay is based on a fluorogenic reaction in which the hydrolyzed peptides with N-terminal free amino-group are converted into FL derivatives in the presence of catechol, borate, and sodium periodate. On FL reaction of peptides with catechol in the presence of borate, the FL product was a complex compound in which boron coordinated to hydroxyl groups of two catechol molecules and N-terminal amino and imino groups in the peptide molecule for emitting fluorescence^8^. On the other hand, N-terminal Ser-containing peptides were selectively derivatized to FL products by catechol with 2-[4-(2-hydroxyethyl) piperazin-1-yl] ethanesulfonic acid instead of borate^11^. The reaction mechanisms and FL products were significantly different between these FL reactions. Thus, the FL reaction using borate was selected to detect the various peptides as enzymatic hydrolyzed products in this research.

This FL reaction is specific for peptides, and any FL derivatives were not produced from other bio-substrates such as amino acids (20 kinds), sugars (glucose and ribose), polyamines (spermine and cadaverine), and nucleic acid bases (adenine, thymine, guanine, and cytosine)^8,11^. Therefore, the enzymatic activities from HCV-PR and HIV-PR in *E. coli* lysate containing many bio-substrates could be detected.

FRET-based assays have been used to determine virus protease activities^6^. These assays employ a FL-labeled peptide as the FRET substrate. The assays are simple and facile, however, the FRET substrates are difficult to design, and noise from the FRET substrate is usually high. The fluorogenic substrates assay and ELISA are also used to determine the proteases activity of the viruses^7,12^. However, these assays need special substrates or antibodies to detect the target protease, and thus, may overlook unexpected virus infection such as an HIV patient co-infected with HCV. On the other hand, the proposed method did not require any specialized labeling of the substrate in advance or antibody, and thus might be applied for simultaneous analyses of multiple enzymatic activities of other proteases. Thus, the proposed method will be a useful tool for treatment and evaluation of medicinal development against viral infections.

In this study, purified HCV-PR and crude HIV-PR expressed in *E. coli* was used. Further studies are necessary to apply the proposed method to clinical samples, i.e. serum from patients co-infected with HCV and HIV. We continue experiment for clinical application of the proposed method.

## Methods

### Materials

HCV-PR was purchased from AnaSpec (USA). Recombinant HIV-PR in *E. coli* lysate was prepared according to previously reported conditions^9^, and was used as a source of HIV-PR. Peptides (SMSYTK, LETSLE, Ac-DTEDVVCCSMSYTK, and Ac-SGIFLETSLE) were purchased from Sigma-Genosys Japan (Japan). The peptides were dissolved in water, and their stock solutions (2.5 mM) were stored at −20 °C.

### Assay of HCV-PR and HIV-PR activities

A typical enzymatic reaction was conducted as follows: a portion of 6 μL of HCV-PR (100 ng) and/or recombinant HIV-PR in *E. coli* lysate was mixed with 2 μL of 500 mM acetate buffer (pH 6.0), and the enzymatic reaction was initiated by adding 6 μL of 2.0 mM HCV-PR substrate (Ac-DTEDVVCCSMSYTK) and 6 μL of 1.0 mM HIV-PR substrate (Ac-SGIFLETSLE)^6,13^. The reaction mixture was incubated at 37 °C for 1 h. Thereafter, each product generated from the substrates was determined by the following FL reaction: a portion of the enzymatic reaction mixture (10 μL) was successively mixed with 5 μL of 5.0 mM catechol, 5 μL of 0.3 M H_3_BO_3_-NaOH (pH 7.0), and 5 μL of 2.5 mM sodium periodate. The mixture was immediately heated at 100 °C for 5 min. This heating was necessary for stopping the enzymatic reaction. After heating, the reaction mixture was cooled in an ice-water bath to stabilize the fluorescent product. The reaction mixture (5 μL) was subjected to HPLC with a spectrofluorometer (RF-10AXL type; Shimadzu, Japan) at 500 nm (emission) with excitation at 400 nm. The fluorogenic products were eluted with 35% methanol and 5% 0.25 M H_3_BO_3_-NaOH (pH 7.0) for 50 min. The HPLC system consisted of a gradient pump (L-6200; Hitachi, Japan) and a reversed-phase column (ODS-100S RP-18e type, 150 mm × 4.6 mm i.d. pore size 5 mm; Tosoh, Japan). All measurements were performed in triplicate for each experiment.

## Supplementary information

Supplementary dataset

## Data Availability

The materials and protocols are available to the public.
