# Supplementary material for: Simultaneous assay for protease activities of hepatitis C virus and human immunodeficiency virus based on fluorescence detection
Source: Sci Rep. 2019 Jun 24;9:9150. doi: 10.1038/s41598-019-45711-0 (PMC6591172; doi:10.1038/s41598-019-45711-0)
Supplement: Supplementary file 1 — Supplementary dataset [file 41598_2019_45711_MOESM1_ESM.pdf]

Simultaneous assay for protease activities of hepatitis C virus and human immunodeficiency virus based on fluorescence detection

Tsutomu Kabashima[1], Keiko Tonooka[2], Makoto Takada[1], Masaaki Kai[3] & Takayuki Shibata[4]

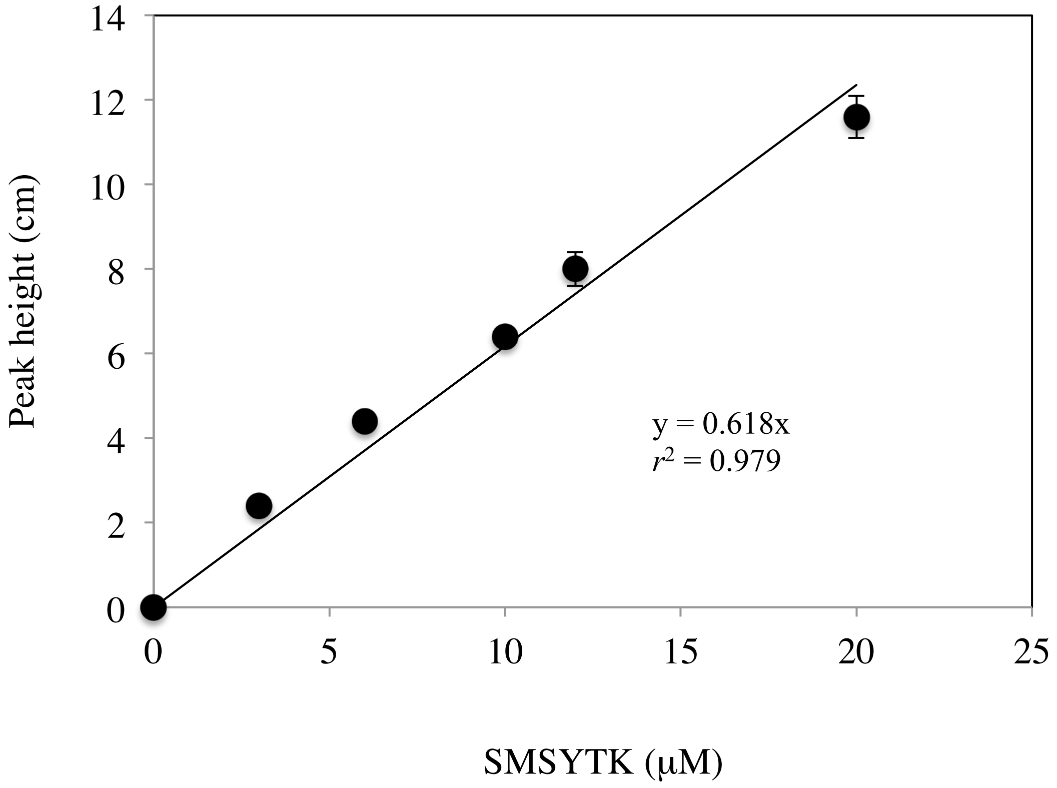

Suppl. Fig. 1. Calibration curve of SMSYTK.

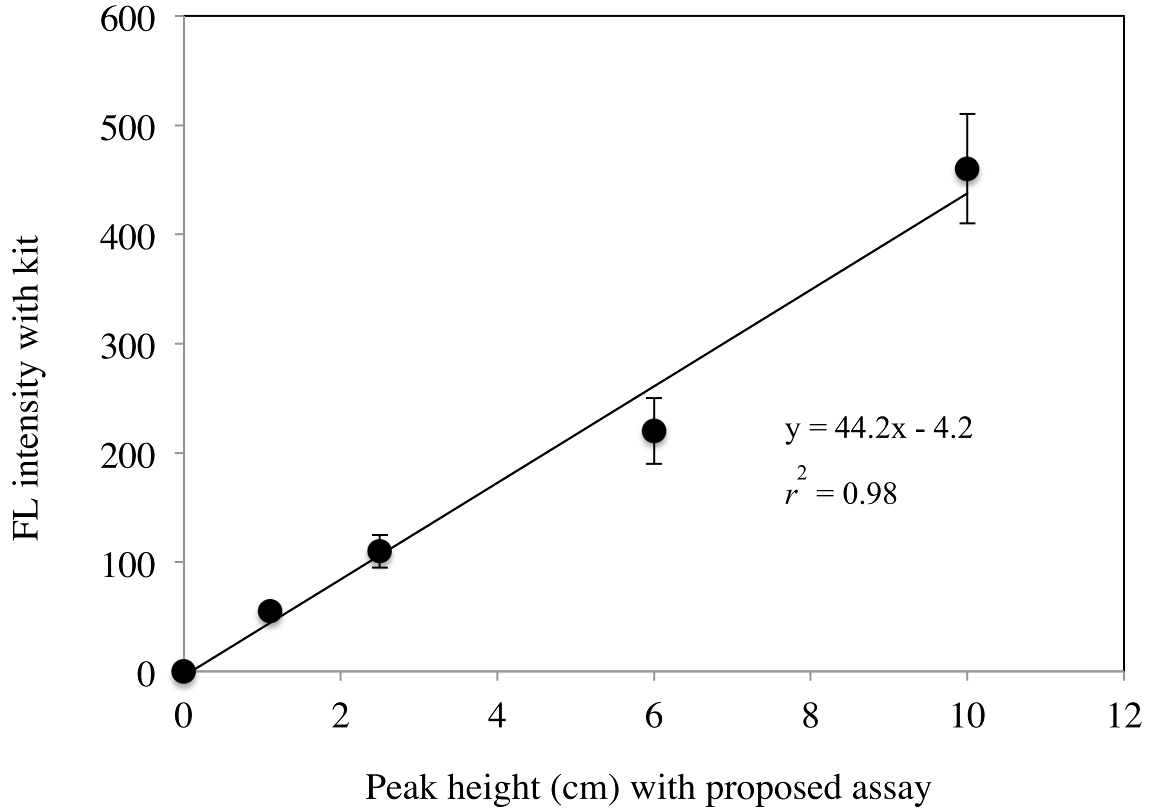

Suppl. Fig. 2. The HCV-PR activity was measured with either the proposed method or a commercial FRET assay kit.

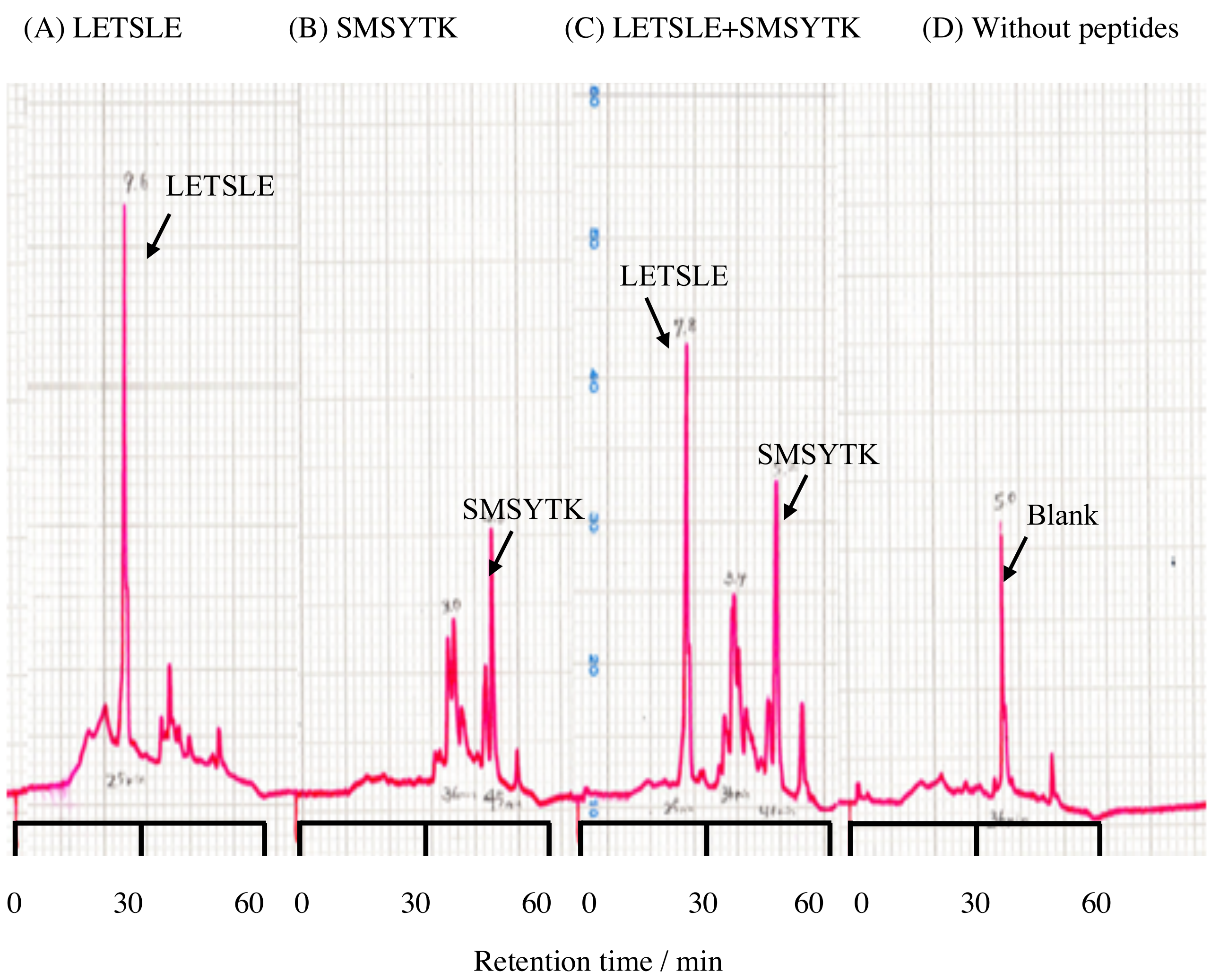

Suppl. Fig. 3. HPLC chromatogram of synthetic peptides. LETSLE and/or SMSYTK were converted to fluorescent derivatives at pH 7.0, and fluorometrically detected with HPLC.
